# Three-Dimensional Evaluation of Impacted Mandibular Canines and Adjacent Structures Using Cone Beam Computed Tomography: A Retrospective Study

**DOI:** 10.3390/jcm14186372

**Published:** 2025-09-10

**Authors:** Ayhan Dogan, Filiz Uslu, Suayip Burak Duman

**Affiliations:** 1 Orthodontics, Aydent Oral and Dental Health Clinic, 44100 Malatya, Turkey; dtayhandogan@gmail.com; 2 Department of Orthodontics, Faculty of Dentistry, Alanya Alaaddin Keykubat University, 07400 Antalya, Turkey; 3 Department of Oral and Maxillofacial Radiology, Faculty of Dentistry, Inonu University, 44280 Malatya, Turkey; suayipburakduman@gmail.com; 4 Department of Diagnostic Sciences, Texas A&M College of Dentistry, Dallas, TX 75226, USA

**Keywords:** arch length, CBCT, impaction, mandibular canine, root resorption

## Abstract

**Objectives:** This study aimed to evaluate impacted mandibular canines and adjacent dentoalveolar structures using cone beam computed tomography. **Methods:** Measurements were made on CBCT images of 54 impacted mandibular canines. Parameters such as the position of the canine teeth, follicle diameter, their relationship with the mental foramen and the incisive mandibular canal, resorption in the adjacent teeth, interpremolar and intermolar width, arch symmetry, and mesiodistal width of the canine teeth were investigated. **Results:** The resorption rate in adjacent permanent teeth was recorded as 14.8%. No statistically significant difference was found between the follicle size of the canine tooth and the resorption in the adjacent teeth (*p* > 0.05). Cortical bone perforation was found in 83.3% of individuals with impacted mandibular canines. Teeth in contact with the mental foramen and incisive mandibular canal were found in 11.1% and 6.5%, respectively. There was no statistically significant difference in the interpremolar and intermolar width on the impacted and non-impacted sides (*p* > 0.05). However, a significant difference was observed in the mesiodistal width of the canines and arch length on the impacted and non-impacted sides (*p* < 0.05). **Conclusions:** No correlation was found between root resorption in adjacent teeth and follicle size. Cortical bone perforations were observed in most impacted canines, and some were in contact with the mental foramen and the incisive mandibular canal. Although transversal arch widths were similar on the impacted and non-impacted sides, differences in arch length and mesiodistal widths may affect arch symmetry and should be considered in treatment planning.

## 1. Introduction

Impacted teeth are defined as teeth that have either partially erupted or are delayed in erupting entirely, resulting in them being unable to function in their natural position and often necessitating treatment [1,2]. Factors contributing to mandibular canine impaction include atypical formations, misalignment of the dental lamina during embryogenesis, endocrine gland dysfunction, genetic factors, buccal inclination of the mandibular incisors, mandibular fractures, premature extraction or retention of deciduous teeth, dental crowding, excessive mesiodistal width of the mandibular canine, odontomas, cysts, adjacent teeth with abnormal morphology, and local factors such as residual root remnants [3,4,5,6]. Impacted teeth can remain in the jawbone for years without inducing pathological issues; nevertheless, they can also lead to aesthetic and functional complications. Moreover, they may result in temporomandibular joint disorders, neuralgiform pain, infections, root resorption of neighboring teeth, migration, arch length reduction, dentigerous cysts, and ameloblastic fibroma [7,8]. For both functional and aesthetic reasons, it is critical to keep the maxillary and mandibular canines in their natural positions. Research on impacted maxillary canines indicates a prevalence ranging from 0.8% to 3% among various ethnicities [9,10,11,12]. The incidence of impacted maxillary canines is considered to be 20 times greater than that of mandibular canines [13].

The prevalence, causes, and treatment outcomes of impacted mandibular canines vary widely. A systematic review indicated that the incidence of impacted mandibular canines is between 0.92% and 1.35%. Nevertheless, research on impacted mandibular canines is constrained by restricted sample sizes and/or specific demographics [3]. Impacted maxillary canines are associated with hazards such as root resorption of adjacent teeth, cyst formation, and the tooth’s inability to erupt in its proper position due to an ectopic eruption [14]. Although comparable risks for impacted mandibular canines may be noticed, the literature has not sufficiently examined the etiological causes and related risk factors. This shortcoming is probably attributable to the usage of panoramic radiographs in prior research for diagnosis and analysis. Research utilizing three-dimensional radiographic imaging seems inadequate [15]. The objectives of this retrospective study, utilizing cone beam computed tomography (CBCT) scans of unilaterally impacted mandibular canines, were as follows:

To determine the location of the impacted mandibular canines.

To determine the association with anatomical features such as the mental foramen and incisive canal.

To assess resorption in adjacent teeth.

To assess and compare the arch symmetry, transversal width, and mesiodistal width of the canine on the impacted and non-impacted sides.

This study hypothesizes that dental arch symmetry is disrupted in individuals with impacted mandibular canines. Specifically, in the presence of an impacted canine, asymmetry is expected, with differences in the mesiodistal widths of the impacted and unimpacted canines and a decreased arch length.

## 2. Materials and Methods

Approval from the ethics committee for this study was obtained by the Inonu University Health Sciences Non-Interventional Clinical Research Board (decision number 2021-1580, dated 26 January 2021). The cone beam computed tomography records of 2500 patients, obtained for treatment reasons at the Department of Oral and Maxillofacial Radiology, Inonu University Faculty of Dentistry, from 2016 to 2021, were analyzed.

The CBCT images included in the study were selected according to the following inclusion criteria: the existence of a unilaterally impacted canine in the mandible, the images being of sufficient quality, the lack of structural defects or syndromes that may impact the affected region, the patients being aged 12 years or older, and the absence of crown-bridge restorations. The exclusion criteria included the patient having received orthodontic treatment, the images being insufficient and of poor quality, having any missing or supernumerary teeth, and the presence of impacted teeth in the lower jaw except the third molar and the lower jaw canine.

This study was conducted using CBCT images from 54 patients (34 females and 20 males) who met the selection criteria among 83 patients with impacted mandibular canines. Radiographic images were obtained with a Newtom 5G (Newtom QR DVT 9000 Quantitative Radiology, Verona, Italy) flat-panel CBCT device. The device operated at 110 kVp with a tube-current range of 1–20 mA under automatic exposure control. The total scan time was 18 s, with a 360° rotation using a cone beam geometry. The acquisition isotropic voxel size was 0.2–0.25 mm. The field of view (FOV) was 18 × 16, 15 × 12, and 12 × 8 mm. Before acquisition, the system performed automatic dose adjustment based on the patient’s bone density. All scans were acquired with patients in the supine position; the head was stabilized and oriented so that the Frankfort horizontal plane was parallel to the gantry and perpendicular to the floor, with the teeth in maximum intercuspation. Patients were instructed to avoid motion during acquisition; scans with motion artifacts were excluded/repeated by institutional protocol. Reconstruction and analysis volumes were reconstructed and reviewed with NNT software (Version 2.21, Verona, Italy). A panoramic image was obtained from the CBCT recording for pre-measurement control. The CBCT images were evaluated using NNT software (Verona, Italy), using 0.2 mm axial, coronal, and sagittal slices. Measurements were made from MPR (multiplanar reconstruction) images with a voxel value of 0.2 mm.

**Evaluation of the angulation of the impacted canine:** On panoramic radiographs obtained from CBCT images, the angulation of the teeth was determined as mesioangular, distoangular, vertical, and horizontal [16].

**Figure 1:**
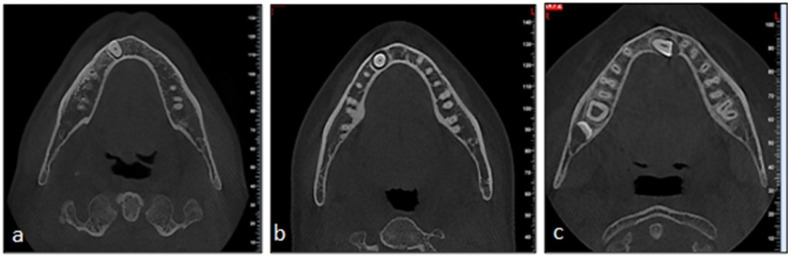
The position of the impacted canine tooth. (**a**) Labial. (**b**) Medial. (**c**) Lingual.

**Evaluation of the labiolingual position of the impacted canine:** In axial section images, the labial, medial, and lingual location of the impacted mandibular canine within the alveolar bone was determined (Figure 1). The relationship with the cortical bone was also examined, and the presence or absence of perforation was assessed.

**Evaluation of the vertical position of the impacted canine:** The vertical position relative to the adjacent incisor was determined at five levels using the cusp of the impacted mandibular canine on the coronal section as a simple and reliable method [17].

- Type 1: Above the cementoenamel junction of the adjacent teeth.
- Type 2: At the height of the cervical third of the adjacent tooth roots.
- Type 3: At the height of the middle third of the adjacent tooth roots.
- Type 4: At the height of the apical third of the adjacent tooth roots.
- Type 5: Below the apices of the adjacent tooth roots.

**The relationship of the impacted canine tooth to the mental foramen was examined** [13]:

1. There is contact with the mental foramen.
2. There is no contact with the mental foramen.

**The relationship of the impacted canine tooth to the incisive mandibular canal was examined** [13]:

1. There is no incisive mandibular canal.
2. There is an incisive mandibular canal, but there is no contact.
3. There is an incisive mandibular canal, and there is contact.

**Evaluation of resorption caused by impacted canines on permanent teeth:** The classification defined by Ericson and Kurol was used to evaluate resorption on adjacent teeth in axial and coronal section images [18]. The Ericson and Kurol scale is a validated and widely accepted grade for the extent of resorptive defects for assessing root resorption.

(0) No resorption: root surface intact.
(1) Slight resorption: root resorption extending to the pulp up to half the dentin thickness.
(2) Moderate resorption: root resorption extending to more than half the pulpal distance, with the pulp margin preserved.
(3) Severe resorption: root resorption reaching the pulp.

**Evaluation of the relationship between the impacted canine and the deciduous canine and resorption** [14,19]:

1. No deciduous mandibular canine.
2. No contact between the permanent and deciduous mandibular canines; no root resorption of the deciduous mandibular canine.
3. There is contact between the permanent and deciduous mandibular canines, but no root resorption of the deciduous mandibular canines.
4. There is no contact between the permanent and deciduous mandibular canines, but there is root resorption of the deciduous mandibular canines.
5. There is contact between the permanent and deciduous mandibular canines, and there is root resorption of the deciduous mandibular canines.

**Figure 2:**
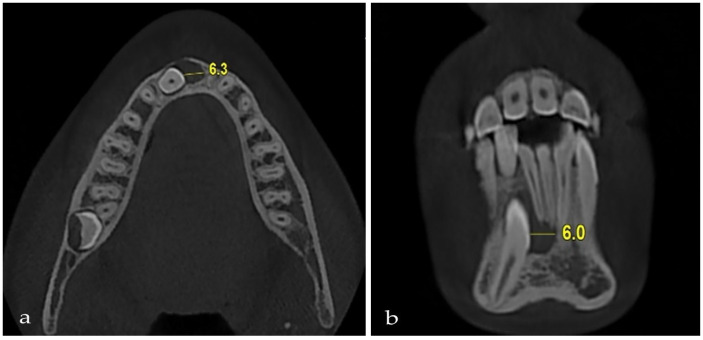
Appearance of follicle diameter. (**a**) Axial section. (**b**) Coronal section.

**Measuring the follicle diameter of the impacted mandibular canine:** This was measured in axial section images at the widest point of the follicle, using a perpendicular line from the follicle wall to the surface of the impacted tooth. The follicle diameter was categorized as either less than 3 mm or greater than 3 mm (Figure 2).

**Figure 3:**
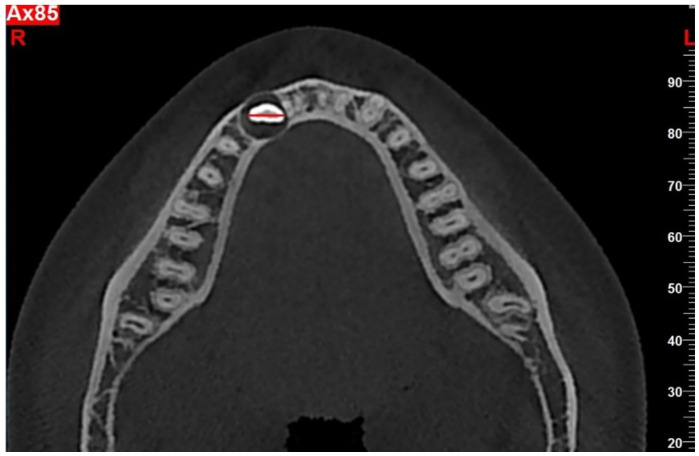
Measurement of the mesiodistal crown width of the impacted canine.

**Measuring the mesiodistal width of the impacted mandibular canine:** This was measured in axial section images at the widest point of the canine (Figure 3).

**Figure 4:**
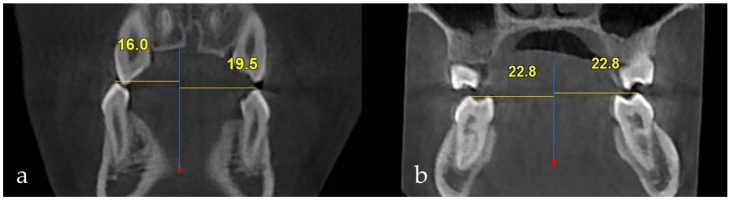
(**a**) Interpremolar width. (**b**) Intermolar width.

**Interpremolar and intermolar widths on the impacted and non-impacted sides were measured:** The mandibular midline was established using the mandibular symphysis, lingual foramen, mental spine, and mental protuberance as reference anatomical landmarks on the sagittal plane [20,21,22,23]. The distance from the buccal cusp of the right and left first premolars and the mesiobuccal cusp of the first molars to the mandibular midline was measured (Figure 4).

**Figure 5:**
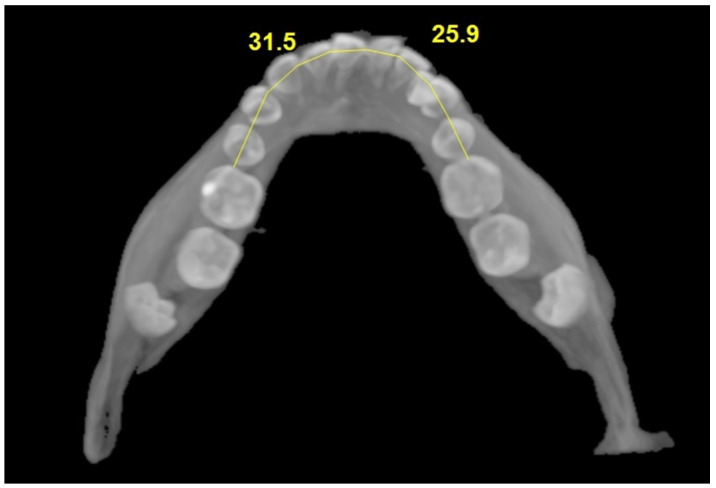
Comparison of arch lengths.

**Arch length measurements on the impacted and non-impacted sides were made:** Arch length measurements were performed directly within the NNT imaging software, without exporting data to any external modeling program. Using the curved planar reformation (panoramic) tool, we defined an occlusal arch curve by placing control points from the mesial surface of the central incisor toward the mesial surface of the first molar on the same side. NNT generated the corresponding curved reformation and reported the cumulative length of this user-defined curve (polyline), which we recorded as the arch length for the impacted and non-impacted sides (Figure 5). Measurements were performed on 0.2 mm MPR reconstructions and recorded in millimeters to the nearest 0.1 mm.

### 2.1. Statistical Analyses

The sample size for the study was calculated utilizing G*Power software (version 3.1.9.7; Franz Faul, University of Kiel, Kiel, Germany). The power analysis, with a 95% confidence level (α = 0.05), 90% power (β = 0.10), and an effect size of 0.45, determined the minimal sample size to be 53. Statistical analyses and calculations were conducted using IBM SPSS Statistics for Windows (Version 21.0, IBM Corp, Armonk, NY, USA).

The distribution of variables, including gender, impacted tooth status, tooth location, deciduous canine status, permanent tooth resorption, etc., was displayed using number (*n*) and percentage (%) data.

The conformity of continuous variables such as interpremolar width, intermolar width, mesiodistal width, and arch lengths to a normal distribution was assessed using the Shapiro–Wilk test. All variables except interpremolar width were determined to be normally distributed. The independent samples *t*-test was used to compare intermolar width, mesiodistal width, and arch lengths, and the Mann–Whitney U test was used to compare interpremolar width. The relationship between canine follicle diameter and resorption in the adjacent permanent tooth was examined using the Chi-square test. Statistical significance was accepted as *p* < 0.05.

To evaluate the factors influencing the arch length (mm) at the impacted side, a multivariable linear regression analysis was conducted. Independent variables included age, mesiodistal width of the canine, interpremolar width, intermolar width, vertical position, location, and angulation. Assumptions of linear regression (normality, homoscedasticity, independence of errors, and multicollinearity) were assessed visually (histogram, P–P plot, and scatterplot) and statistically (Durbin–Watson test and VIF), and all were met.

### 2.2. Evaluations of Measurement Error

Measurements were performed at a single center by one researcher. To assess the measurement error, 25% of the measurements were re-evaluated 15 days after the initial recordings, and the measurement error was analyzed. The intraclass correlation coefficient (ICC) was used to assess the intra-observer correlation coefficients for each parameter. The intra-observer reliability was 97%.

## 3. Results

In the study, 63.0% (*n* = 34) of individuals were female, whereas 37.0% (*n* = 20) were male. The ages of the participants varied from 12 to 36 years, with a mean age of 17.39 ± 6.10 years. The average age of females was 16.06 ± 4.38 years, while that of males was 19.65 ± 7.88 years. A total of 51.9% (*n* = 28) of the impacted canines were located on the right side, whereas 48.1% (*n* = 26) were located on the left side (Table 1).

Based on the angulation of the impacted canines, 9.3% (*n* = 5) were horizontally positioned, 38.8% (*n* = 21) were mesioangular, 42.6% (*n* = 23) were vertically positioned, and 9.3% (*n* = 5) were distoangular. In the labiolingual position, 51.9% (*n* = 28) of the teeth were identified as labial, 18.5% (*n* = 10) as lingual, and 29.6% (*n* = 16) as medial (Table 2).

In individuals with impacted mandibular canines, 83.3% (*n* = 45) exhibited cortical bone perforations. A total of 64.4% (*n* = 29) of the cortical bone perforations were labial, 26.7% (*n* = 12) were lingual, and 8.9% (*n* = 4) were bilateral. Upon examination of the vertical positioning of the canines in relation to the adjacent incisors, 16.7% (*n* = 9) were classified as type 1, 9.3% (*n* = 5) as type 2, 22.2% (*n* = 12) as type 3, 37.0% (*n* = 20) as type 4, and 14.8% (*n* = 8) as type 5 (Table 2).

It was noted that 11.1% (*n* = 6) of the canines were in contact with the mental foramen, but 88.9% (*n* = 48) were not. Incisive canals were not present in 14.8% (*n* = 8) of individuals, while 85.2% (*n* = 46) had incisive canals. Among individuals with incisive canals, 6.5% (*n* = 3) exhibited contact with the impacted mandibular canine, whereas 93.5% (*n* = 43) had no contact (Table 2).

Permanent tooth resorption occurred in 14.8% (*n* = 8) of the individuals who participated in the research. Among the canines responsible for permanent tooth resorption, 62.5% (*n* = 5) were labial, 25.0% (*n* = 2) were lingual, and 12.5% (*n* = 1) were medial. No resorption was detected in any permanent premolars; nevertheless, 75% (*n* = 6) of the resorbed teeth were lateral incisors, while 25% (*n* = 2) were central incisors. Resorption was observed in the middle third of the root in 50% of the affected teeth, in the apical third in 37.5%, and in the cementoenamel junction in 12.5%. Upon evaluating the severity of resorption, moderate resorption was identified in 50%, slight resorption in 37.5%, and severe resorption in 12.5% (Table 2) (Table 3).

Of the individuals studied, 55.6% (*n* = 30) had deciduous canine teeth, whereas 44.4% (*n* = 24) did not. A total of 53.4% (*n* = 16) of individuals with deciduous canine teeth exhibited resorption with follicle contact, while 20.0% (*n* = 6) showed resorption without follicle contact. Additionally, 3.3% (*n* = 1) had no resorption but presented with follicle contact, and 23.3% (*n* = 7) had neither resorption nor follicle contact (Table 2).

Among the canine teeth, 75.9% (*n* = 41) exhibited follicle diameters less than 3 mm, whereas 24.1% (*n* = 13) displayed follicle diameters greater than 3 mm. Permanent tooth resorption occurred in 14.6% (*n* = 6) of teeth with a follicle diameter less than 3 mm, whereas it was observed in 15.4% (*n* = 2) of teeth with a follicle diameter greater than 3 mm. A statistically significant difference was not observed in the presence or absence of permanent tooth resorption relative to follicle diameter (χ2 = 0.004, *p* = 0.947) (Table 2) (Table 4).

The mean mesiodistal width of the impacted canine was 6.78 ± 0.41, while that of the non-impacted canine was 6.56 ± 0.40. A statistically significant difference in mesiodistal widths was observed (t = 2.617, *p* = 0.010). A statistically significant difference was also observed in the arch lengths between the impacted and non-impacted canine sides in relation (t = 5.194, *p* < 0.001). The arch length on the impacted side of the tooth was shorter than that on the non-impacted side. No statistically significant difference was observed between the interpremolar and intermolar widths (*p* > 0.05) (Table 5).

Table 6 summarizes the regression coefficients, 95% confidence intervals, and *p*-values. The overall regression model was statistically significant (F = 3.356, *p* = 0.002), with an explained variance of R^2^ = 0.522 and adjusted R^2^ = 0.366. In the multivariable linear regression analysis, intermolar width and tooth angulation were significant predictors of the arch length. Each one-millimeter increase in intermolar width was associated with a 0.57 mm increase in the arch length (B = 0.570, 95% CI: 0.127–1.013, *p* = 0.015). With respect to angulation, canines in a horizontal position demonstrated an average arch length that was 2.88 mm shorter compared with mesioangular canines (B = −2.884, 95% CI: −5.003 to −0.765, *p* = 0.011).

Other predictors, including age (B = −0.047, 95% CI: −0.137–0.043, *p* = 0.312), mesiodistal width of the canine (B = −0.391, 95% CI: −1.781–0.999, *p* = 0.584), and interpremolar width (B = 0.204, 95% CI: −0.168–0.576, *p* = 0.290), did not show statistically significant associations with arch length. Similarly, vertical position (types 1–3 and 5, reference type 4), location (lingual and medial, reference labial), and other angulation categories (vertical and distoangular) were not significant (all *p* > 0.05), as shown in Table 6.

## 4. Discussion

An impacted tooth is defined as a tooth that is not correctly positioned within the dental arch, resulting from delays during the eruption phase of normal development [11,24]. CBCT images were obtained of a unilaterally impacted mandibular canine, with the contralateral canine positioned in occlusion or near it. A split-mouth study was conducted to compare the dentoalveolar structures of impacted and non-impacted canines within the same patient [25,26].

Studies indicate a higher incidence of impacted mandibular canines in both males [13] and females [15,27,28]. Walker et al. [12] indicated that the prevalence of impacted maxillary canines is higher in women, possibly due to genetic factors and variations in craniofacial growth and development between genders. Another reason could be that women exhibit more interest in orthodontic treatment compared to men [12,17]. The study found a higher prevalence of impacted mandibular canines in women.

Studies show that there is no significant difference between the presence of impacted mandibular canines on the right or left side [16,27,29]. In this study, the distribution of impacted mandibular canines on the right and left sides was almost equal. Because the sample sizes in these studies are limited, further research with larger participant groups is necessary.

Yavuz et al. [16] found that in panoramic radiographs, the angulation of impacted mandibular canines was observed as follows: 32.4% in the mesioangular position, 8.5% in the distoangular position, 40.8% in the vertical position, and 18.3% in the horizontal position. Our study revealed a higher frequency of impacted mandibular canines in vertical and mesioangular positions.

Many studies indicate that impacted maxillary canines are positioned palatally [12,17,18,30]. Impacted mandibular canines are most frequently positioned labially [13,15,27,29,31]. Our study identified the most common location for impacted mandibular canines as labial, followed by medial, with lingual being the least common.

The vertical position of impacted canines relative to adjacent incisors was generally reported in the cervical, middle, and apical thirds [13,15]. Our study showed the following distribution: 16.7% in the cervical third, 22.2% in the middle third, 37.0% in the apical third, 14.8% below the apex, and 9.3% at the cementoenamel junction. Yavuz et al. [16] evaluated the vertical position of impacted mandibular canines in panoramic images and found their positions to be between the crown and cervical margin of the adjacent incisor (15.5%), between the cervical margin and the apex of the tooth (46.5%), and below the apex of the adjacent tooth (38%).

Karabaş et al. [13] reported a rate of 13.6% for impacted canines in contact with the mental foramen, whereas our study found a rate of 11.1%. The examination of the incisive canal revealed a contact/proximity rate of 62.1% between the impacted tooth and the incisive canal. The contact rate between the impacted canine and the incisive canal was recorded as 6.5% in our study. The exclusion of teeth not in contact with the incisive canal may have contributed to the discrepancies observed in the results of our study. The proximity of impacted mandibular canines to the incisive canal and mental foramen has important diagnostic and surgical implications. Examining these areas with CBCT may be advantageous in preventing neurovascular injuries and other postoperative complications [32,33]. Precautions should be taken during surgical procedures involving the mandibular canal and mental foramen. Careful flap design, minimal bone removal, and meticulous tissue management in cases with proximity to the mental foramen are important to reduce the risk of postoperative complications [32,33].

Root resorption was evaluated using the Ericson and Kurol scale, a widely accepted and frequently referenced method in the literature for grading incisor root resorption associated with impacted canines. Ericson and Kurol [18] reported that resorption of maxillary ectopic canines on adjacent incisors was 50% more detectable when assessed with CT. They stated that this was due to the inadequate observation of root morphology on conventional radiographs and the superimposition of the incisor roots on the crown of the impacted canine. The presence of resorption in adjacent teeth associated with impacted mandibular canines has been documented at rates of 7.3% and 18.1% [13,15]. Our study found that root resorption only occurred in the incisors and was not seen in the premolars. This finding is consistent with CBCT data showing that mandibular canine impaction causes a low rate of nearby tooth resorption, which is described as uncommon and mild and usually does not involve the premolars [13,15]. The distribution of resorptive findings is explained by the anatomical pathway of eruption, which is usually buccal or lingual, and the lack of direct contact with the premolar roots. The presence of resorption should be assessed before orthodontic treatment planning. Numerous researchers have investigated the relationship between follicle size and resorption in impacted maxillary canines [12,19,34]. These studies indicated no correlation between follicle size and permanent tooth resorption. Upon analyzing the correlation between the follicle size of impacted mandibular canines and permanent tooth resorption, no statistically significant difference was identified, similar to the findings observed in maxillary canines.

In our study, the rate of deciduous canine teeth remaining in the mouth was recorded as 55.6%. There may be a problem with the eruption of the permanent canine teeth due to the presence of a persistent deciduous canine.

There have been studies published that compare the mesiodistal widths of impacted and non-impacted maxillary canines [35,36]. However, no similar study has been found on impacted mandibular canines. In our study, the mean mesiodistal width of the impacted mandibular canine was found to be 6.78, and the mean mesiodistal width of the non-impacted canine was found to be 6.56. A statistically significant difference was found between the mesiodistal widths of the impacted and non-impacted canines. This result is similar to the results of studies in the literature reporting that the mean mesiodistal width of impacted maxillary canines is greater than the mean mesiodistal width of the non-impacted canine [35,36,37]. The increased mesiodistal width mentioned in impacted mandibular canines may have significant etiological effects. Increased mesiodistal width may be a contributing etiological factor for impacted mandibular canines, as suggested by studies on maxillary canines [35,36,37].

Some studies on impacted maxillary canines have evaluated the interpremolar and intermolar distances to measure transversal width. Al-Nimri and Gharaibeh [38] reported that the interpremolar and intermolar arch widths on the impacted side of the maxillary canines were significantly greater than those on the non-impacted side. In another study, the width in the premolar region was found to be smaller on the impacted canine side of the maxilla than on the non-impacted side [25,39]. Langberg and Peck [40] reported that the interpremolar and intermolar arch widths were similar on the impacted and non-impacted sides. In our study, similar interpremolar and intermolar distances were noted between the impacted and non-impacted sides.

Bertl et al. [15] reported that 44.6% of canines contacted cortical bone in their study, while Karabaş et al. [13] reported this rate as 40.9%. In contrast to these studies, ours revealed a higher rate of contact between impacted canine teeth and the cortical bone. The reason for the high number of teeth contacting cortical bone in our study may be that the measurements were made at the crown level, whereas in other studies, the measurements were made at the apex of the canine. We believe that this high rate of perforation in cortical bone is due to the limited buccolingual width of the alveolar bone. This is not only due to methodological differences but can also have important clinical implications. Alveolar bone loss and cortical bone perforation increase the risk of surgical complications and may complicate the orthodontic treatment of impacted canines. They may also increase the risk of periodontal problems, including gingival recession and alveolar defects. These complications may require more surgeries and longer treatment periods. Therefore, three-dimensional assessment is essential for effective treatment planning and risk management.

There are studies in the literature that measured the arch length in patients with impacted maxillary canines using models and scanning materials [38,41,42,43]. Another study reported a difference in arch length between the sides at both the impacted and non-impacted canines, with the arch length being shorter on the impacted canine side [39]. No study was found that measured arch length using CBCT images of impacted mandibular canines. In our study, the arch length of the impacted canine side was found to be shorter than the arch length of the non-impacted canine side. The eruption of the canine is an important factor in increasing anterior segment length [44]. The analyses showed that the arch length was primarily affected by intermolar width and tooth angulation. As intermolar width increased, the arch length also increased, while the arch length decreased in horizontally positioned canines compared to mesioangularly positioned canines. No significant effects were found for other positional variables.

The study identified a relatively high number of impacted mandibular canines; however, further research with larger sample sizes and multicenter CBCT studies is necessary. Regular clinical and radiological examinations, including CBCT, are advised during the mixed dentition phase to facilitate early diagnosis and enhance treatment planning accuracy.

## 5. Conclusions

The mesiodistal width of the impacted canine exceeded that of the non-impacted ca-nine. The arch length was found to be shorter on the impacted canine side, whereas the interpremolar and intermolar distances were comparable on both sides. Evaluation of these parameters is essential prior to orthodontic treatment planning.

## Figures and Tables

**Table 1:** Gender, age, and impaction status of individuals with impacted canine teeth.

	*n* (%)	Mean ± SD	Median (IQR)	Min; Max
Gender				
Female	34 (63.0)	16.06 ± 4.38	15.0 (5.0)	12.0; 35.0
Male	20 (37.0)	19.65 ± 7.88	17.0 (11.8)	12.0; 36.0
Impacted tooth:				
Right mandibular canine	28 (51.9)			
Left mandibular canine	26 (48.1)			

IQR—interquartile range.

**Table 2:** Radiographic assessment of impacted mandibular canines: positional and anatomical features.

		*n* (%)
Angulation of the canine tooth	Horizontal	5 (9.3)
	Mesioangular	21 (38.8)
	Vertical	23 (42.6)
	Distoangular	5 (9.3)
Labiolingual position of the canine crown	Labially impacted	28 (51.9)
	Lingually impacted	10 (18.5)
	Medially impacted	16 (29.6)
Vertical position of the canine cusp tip	Type 1	9 (16.7)
	Type 2	5 (9.3)
	Type 3	12 (22.2)
	Type 4	20 (37.0)
	Type 5	8 (14.8)
Contact with the mental foramen	Yes	6 (11.1)
	No	48 (88.9)
Incisive mandibular canal	Yes	46 (85.2)
	No	8 (14.8)
Contact with the incisive mandibular canal	Yes	3 (6.5)
	No	43 (93.5)
Resorption of adjacent permanent tooth	No	46 (85.2)
	Slight resorption	3 (37.5)
	Moderate resorption	4 (50.0)
	Severe resorption	1 (12.5)
Localization of resorption	Cementoenamel junction	1 (12.5)
	Apical third	3 (37.5)
	Middle third	4 (50.0)
Deciduous mandibular canine	No	24 (44.4)
	Yes	30 (55.6)
Resorption and contact in deciduous canine	Contact, resorption	16 (53.4)
	No contact, resorption	6 (20.0)
	Contact, no resorption	1 (3.3)
	No contact, no resorption	7 (23.3)
Cortical bone perforation	No	9 (16.7)
	Labial	29 (64.4)
	Lingual	12 (26.7)
	Labial and lingual	4 (8.9)
Follicle diameter	<3 mm	41 (75.9)
	≥3 mm	13 (24.1)

**Table 3:** Distribution of permanent tooth resorption according to the position of the canine tooth.

	Adjacent Permanent Tooth Resorption	
	No *n* (%)	Yes *n* (%)
Location		
Labial	23 (50.0)	5 (62.5)
Lingual	8 (17.4)	2 (25.0)
Medial	15 (32.6)	1 (12.5)

**Table 4:** Distribution of adjacent permanent tooth resorption according to follicle diameter.

	Adjacent Permanent Tooth Resorption			
	No *n* (%)	Yes *n* (%)	Test Statistic	
			χ^2^	*p*
Follicle diameter				
<3 mm	35 (85.4)	6 (14.6)	0.004	0.947
>3 mm	11 (84.6)	2 (15.4)		

χ^2^ = Chi-square test statistic.

**Table 5:** Comparison of measurements on the impacted and non-impacted sides of the canine tooth.

	Impacted Side	Non-Impacted Side	Test Statistic	
	Mean ± SD Median (IQR)	Mean ± SD Median (IQR)	z; t	*p*
Mesiodistal width of the canine	6.78 ± 0.41	6.56 ± 0.40	t = 2.617	0.010
	6.70 (0.50)	6.60 (0.50)		
Interpremolar width	15.69 ± 2.29	16.45 ± 1.42	z = 1.864	0.062
	16.00 (2.78)	16.80 (1.65)		
Intermolar width	21.86 ± 2.27	22.44 ± 2.06	t = 1.393	0.166
	22.15 (2.90)	22.35 (2.80)		
Arch length	29.92 ± 2.38	32.21 ± 2.18	t = 5.194	<0.001
	30.05 (2.80)	32.40 (3.00)		

t: independent samples *t*-test; z: Mann–Whitney U test; IQR: interquartile range.

**Table 6:** Results of multivariable linear regression analysis assessing factors associated with the arch length (mm) at the impacted side.

Variable	B (95% CI)	SE	*p*-Value
Age	−0.047 (−0.137, 0.043)	0.046	0.312
Mesiodistal width	−0.391 (−1.781, 0.999)	0.709	0.584
Interpremolar width	0.204 (−0.168, 0.576)	0.19	0.290
Intermolar width	0.570 (0.127, 1.013)	0.226	0.015
Vertical position: type 1 (ref = type 4)	0.320 (−2.003, 2.643)	1.152	0.784
Vertical position: type 2 (ref = type 4)	1.297 (−1.295, 3.889)	1.317	0.329
Vertical position: type 3 (ref = type 4)	0.602 (−1.625, 2.829)	1.123	0.594
Vertical position: type 5 (ref = type 4)	0.206 (−1.528, 1.940)	0.88	0.820
Location: lingual (ref = labial)	0.391 (−1.158, 1.940)	0.787	0.629
Location: medial (ref = labial)	0.603 (−1.214, 2.420)	0.927	0.512
Angulation: horizontal (ref = mesioangular)	−2.884 (−5.003, −0.765)	1.081	0.011
Angulation: vertical (ref = mesioangular)	−0.034 (−2.107, 2.039)	1.041	0.973
Angulation: distoangular (ref = mesioangular)	−0.102 (−2.011, 1.807)	0.952	0.921

## Data Availability

The original contributions presented in this study are included in the article. Further inquiries can be directed to the corresponding author.

## Abbreviations

The following abbreviations are used in this manuscript:

CBCT	Cone beam computed tomography
